# Temporally Resolved Intensity Contouring (TRIC) for characterization of the absolute spatio-temporal intensity distribution of a relativistic, femtosecond laser pulse

**DOI:** 10.1038/s41598-019-42683-z

**Published:** 2019-05-22

**Authors:** Daniel Haffa, Jianhui Bin, Martin Speicher, Klaus Allinger, Jens Hartmann, Christian Kreuzer, Enrico Ridente, Tobias M. Ostermayr, Jörg Schreiber

**Affiliations:** 1 Lehrstuhl für Medizinphysik, Fakultät für Physik, Ludwig-Maximillians-Universität München, 85748 Garching b. München, Germany; 2 Max-Planck-Institut für Quantenoptik, 85748 Garching b. München, Germany; 3 Accelerator Technology and Applied Physics Division, Lawrence Berkeley National Laboratory, Berkeley, CA 94720 USA

**Keywords:** Imaging and sensing, Ultrafast photonics, Laser-produced plasmas

## Abstract

Today’s high-power laser systems are capable of reaching photon intensities up to 10^22^ W cm^−2^, generating plasmas when interacting with material. The high intensity and ultrashort laser pulse duration (fs) make direct observation of plasma dynamics a challenging task. In the field of laser-plasma physics and especially for the acceleration of ions, the spatio-temporal intensity distribution is one of the most critical aspects. We describe a novel method based on a single-shot (i.e. single laser pulse) chirped probing scheme, taking nine sequential frames at frame rates up to THz. This technique, to which we refer as temporally resolved intensity contouring (TRIC) enables single-shot measurement of laser-plasma dynamics. Using TRIC, we demonstrate the reconstruction of the complete spatio-temporal intensity distribution of a high-power laser pulse in the focal plane at full pulse energy with sub-picosecond resolution.

## Introduction

Since the advent of chirped pulse amplification^1,2^ high-power laser systems have evolved and now enable novel particle acceleration techniques. Multi-GeV electrons^3^, x-rays^4^, neutrons^5^ and energetic ions^6,7^ emerge from intense laser-plasma interactions with targets. A milestone for acceleration of multi MeV ions has been demonstrated 2000^8^. Short acceleration lengths (MeV/µm), small source sizes (µm), high particle flux^9,10^, poly-energetic energy distributions and broad angular divergence^11^ mark the key differences compared to the output from conventional accelerators. Substantial improvements of laser and target technology have enabled higher repetition rates also for solid targets^12–16^. With the new generation of petawatt class laser systems^17,18^ proton energies up to 100 MeV^19^ in a single shot and repetition rates up to 1 Hz^20^ at lower kinetic energies are now available, fueling enthusiasm on the way to developing laser-driven systems^11^.

Two of the most influential aspects relevant to laser-ion acceleration are the temporally resolved intensity contrast^21^ and the transverse intensity distribution in the focal plane. Due to sub-picosecond duration and the high peak intensity a direct measurement of such remains challenging. In fact the spatial and temporal intensity distributions are often determined independently, for separate shots and with attenuated beams. The temporal intensity contrast is typically measured relatively (in relation to the peak intensity) with autocorrelation methods^22,23^ which integrate over the spatial distribution. Newest approaches aiming for single-shot measurements^24,25^ seem very promising for monitoring the temporal shape of the intensity during the interaction. The spatial intensity distribution is usually measured with time-integrating beam profilers using an attenuated laser pulse and the anticipated high intensities require mapping with high dynamic range (at least five orders of magnitude) in order to avoid an overestimation of the peak intensity at focus^26^. Also several methods have been developed to measure the peak intensity of high-power laser pulses^27–29^. A combined spatio-temporal measurement of an attenuated high-power laser focus has recently been demonstrated for the first time^30^. Although several techniques allow the analysis of the spatial and temporal intensity distribution of a high-power laser focus^22–26,31,32^, it has not been directly accessible during a laser-plasma interaction. Here we describe a technique that measures the spatio-temporal evolution of a laser-induced plasma on target for a single-shot. This not only yields information about the evolution of plasma formation, but can be further interpreted to retrieve the absolute spatio-temporal intensity distribution (STID) of a high-power laser in the focal plane at full intensity. Within a single-shot we measure temporally resolved intensity contours with sub-picosecond temporal resolution and about 25 µm spatial resolution. With each additional measurement with modified laser intensity (in our example five pulses) we add about one order of magnitude to the covered dynamic range of our measurement. Because there are no direct comparisons with our novel technique, we extract the transverse spatial and the temporal profiles of the three-dimensional intensity distribution function, and compare them to the standard techniques, i.e. a spatial high dynamic range (HDR) focus image (integrated over the full laser pulse duration) and a temporal contrast measurement taken with a third-order autocorrelator.

## Experimental Setups

**Figure 1:**
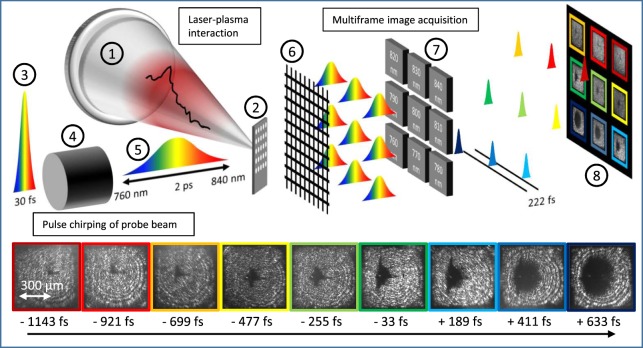
Experimental Setup for TRIC. A Ti:Sapphire laser pulse (1) is focused onto a 200 nm thick Formvar target (2) at 45° incidence. A small part of the short laser pulse is coupled out earlier (3) and sent through a glass rod (4). The emerging chirped pulse (5) passes the target perpendicular to the main pulse. In the imaging path, the probe beam is multiplied using a low dispersion transmission grating (6). A small frequency range is cut out of each of the replicas by narrow bandpass filters (7) before being recorded with a camera (8). The bottom row shows a sample picture series recorded in a single-shot.

The experimental setup resembles a typical pump-probe configuration and is shown in Fig. 1. The Ti:Sapphire system provides 374 mJ on target within 30 fs with a spectral range of 760 nm to 840 nm. The laser is focused onto a 200 nm thick Formvar foil target^15^ with a diameter of 2 mm which is positioned for irradiation at 45 degree incidence. A small fraction of the 30 fs laser beam is coupled out in advance to provide the probe beam. After traversing a glass rod, the resulting chirped probe pulse of 2 ps duration is overlapped perpendicularly with the main laser pulse at the target. The transmitted probe light is collected with a lens, guided from the vacuum chamber and imaged with another lens onto a camera (Prosilica GT 4907, Allied Vision) with a magnification of ≈6.5 and a spatial resolution of roughly 1.5 µm. A 2D grating (Collischon, 15 µm lattice constant) is positioned between the lens and the CCD chip, such that nine spatially separated replicated images are accommodated on the camera chip. Each image still contains the complete spectrum. By adding nine different narrow band-pass filters (bandwidth of 10 nm) in front of the camera chip at the positions of the replicas they will be spectrally filtered and thus correlated temporal information is imprinted^33,34^ onto each image. The time delay between subsequent images is defined by the set spectral chirp and in our case set to 222 fs (see methods). We thus measure nine sequenced images with a frame rate of 5 THz, revealing the plasma evolution in response to a high-power laser pulse interacting with a solid density target. It is worth mentioning, that this implementation can considerably simplify single-shot probe schemes that have been realized in the past^35–38^. An exemplary raw image of such measurement is displayed in Fig. 1. The image appears bright in areas where the target remains transparent and dark in areas, where the target became opaque. The cause for this binary image information is the change in the optical transmission of the evolving plasma. When the free electron density becomes larger than the critical density *n*_*e*_ > *n*_*c*_, the plasma turns reflective for the incoming probe pulse. The horizontal dimension recorded in the image contains convoluted information since the laser and the orthogonal probe pulse both hit the target under an angle of 45 degree. The probe image is thus a projection. At the same time, the orientation of the target with respect to the drive beam implies that non-central parts of the target interact with the laser in out-of-focus planes causing a complex two-fold convolution of time and space. A spatial difference in horizontal dimension of 60 µm in the image, corresponds to a time difference of 200 fs of both the laser and probe pulse hitting the target. For simplicity we therefore only consider the vertical dimension, where those effects do not play a role.

## Relation of Laser Intensity and Plasma Contour

The measured average growth of the overdense plasma area (dark region) of the single-shot image sequence in Fig. 1 is evaluated to be about 30% of the speed of light. The first step was to examine the source for this plasma expansion in more detail.

**Figure 2:**
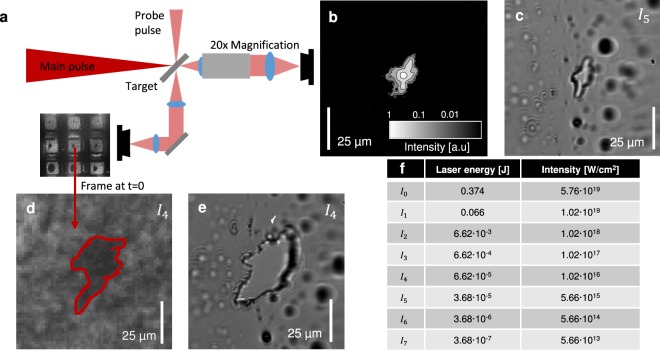
Nexus of intensity distribution and plasma shape. This figure shows separately obtained images of the laser focus, the probe image and the hole in the target after the shot. The configuration can be seen in (**a**), a 20 times magnifying microscope views the target under an angle of 45° and also measures an attenuated pulse in the focal plane (**b)** (contour image). The hole in the target with intensity *I*_5_ is shown in (**c)**. A shot with intensity *I*_4_ compares the hole in the target (**e**) to the probe image (**d)**. The table (**f)** lists the laser energies on target and corresponding intensities used during the experiment.

**Figure 3:**
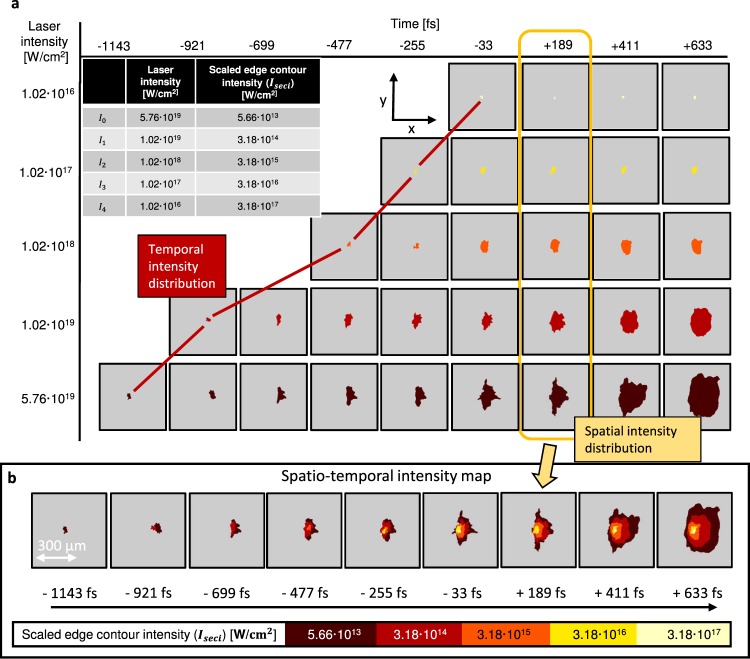
Spatio-temporal intensity distribution. (**a**) shows the scaled edge contours exceeding a certain intensity ${I}_{{\rm{seci}}}$. Each row corresponds to a single shot with a certain laser intensity (attenuation coefficient) of the laser pulse and thus a different intensity of the scaled edge contour line. While the edges exactly have the depicted intensity, the inner part of the enclosed contour cannot be specified. The table shows the used peak laser intensities and the corresponding measured scaled edge contour intensity. The yellow frame shows the spatial distribution at the peak of the laser pulse *t* = 189 fs (first frame after t = 0) and the red line the temporal intensity distribution of *x* = 0 µm and *y* = 15 µm. (**b)** is the absolute spatio-temporal intensity map and is the main result. Each time step represents the summation over a column in (**a)**.

We therefore varied the laser intensity and compared the spatial intensity distribution of the laser focus with the contour of the overdense plasma during the interaction and the hole at the target long time after each shot. The laser energy was reduced by adding neutral density filters in the beam path of the laser before compression and thus attenuating both the main pulse and the probe pulse. The corresponding intensities are shown in Fig. 2f. The hole in the target after the shot with intensity *I*_5_ is shown in Fig. 2c. Since the angle of the target was 45 degree with respect to the laser axis and thus also to the microscope (Fig. 2a), only the central vertical axis appears sharp. Comparison to a contour plot of the laser focus, taken with an attenuated beam (Fig. 2b), indicates great resemblance. The picture was taken with the same microscope. Comparison of the laser intensity profile (Fig. 2b) and the hole in the target after the shot (Fig. 2c) indicates the contour of the hole to be directly correlated to the shape of the laser focus at a certain intensity. The distortion of the laser focus makes this effect more clear. In Fig. 2d we see the measured plasma shape with the probing technique at *t* = 0 (largest contour) with an intensity of *I*_4_ of the main pulse. The hole in the target foil after the shot is shown in Fig. 2e and again the contours in Fig. 2d,e show excellent resemblance. This indicates that the threshold intensity, where the Formvar target becomes opaque and thus *n*_*e*_ ≥ *n*_*c*_ can be related to the contour of the hole in the target after the shot. As a conclusion of this empirical observation we state, that the intensity at the measured contour of the plasma is the isosurface of the intensity distribution of the laser focus reaching a certain threshold intensity. This threshold ${I}_{{\rm{th}}}=\mathrm{(6}\pm \mathrm{5)}\cdot {10}^{13}$ · W cm^−2^ was determined by reducing the laser energy stepwise and thus the intensity until no hole was observed at the target after the shot (see methods). In the previous part we have simplified a few physical aspects. This is mostly valid since the threshold intensity (${I}_{{\rm{th}}}=\mathrm{(6}\pm \mathrm{5)}\cdot {10}^{13}$ · W cm^−2^) is significantly lower than relativistic intensities ≈10^18^ · W cm^−2^, where other effects such as relativistic transparency^39^, relativistic self-focusing^40^ and self-phase-modulation^41^ can play a role. We further assume, that the enlargement of the plasma is caused, when the intensity distribution of the laser pulse reaches the threshold intensity and the target is ionized to reach free electron density beyond the critical density *n*_*e*_ ≥ *n*_*c*_. Another cause of an advancing plasma diameter could be the transverse expansion of the plasma itself by hot electrons^42,43^. In the center of the laser focus at *t* = 0 the laser eventually reaches relativistic intensities for the shots with highest laser energy and thus in the inner part relativistic transparency, self-focusing and other effects can play a significant role. Also the electron temperature at the central part of the laser focus can be beyond the MeV level, resulting in expansion velocities approaching a large fraction of c. However, since the interesting region for TRIC is the outer contour of the plasma (where I(x, y, t) = *I*_th_) those effects do not play a role and the electron temperature is in the low keV region or smaller. In principle, fast electrons that are excited in the center, where the intensity is higher, could contribute to the transversely expanding front. Because in our case the transverse expansion primarily in vertical dimension stagnates after *t* = 0 when the peak of the pulse has hit the target, we ignore this effect but note that it could become relevant for transversely steep intensity distributions. We observed a growth in horizontal dimension after *t* = 0 s for the highest intensities (Fig. 3). One reason for this delayed growth is the orientation of the target, which we irradiate and view under an angle of 45°. Therefore, an intensity contour with transverse extension dx will cause an effect in the target that is delayed by *dx*/*c*, in our case ≈200 fs. In addition, and in particular for spatially narrow intensity distributions, hot electrons that are generated in the center of the plasma at *t* = 0 s will eventually reach the outer ionization border and could cause further expansion at later times. In our case, the plasma growth at the contour edge is assumed to be dominated by the expanding ionization front due to the raising intensity of the laser pulse.

## Results

### Spatio-temporal intensity distribution

The measured contour lines originate when the STID reaches the threshold intensity. The intensity at the edge contour is thus

1 $$I(x,y,t)={I}_{{\rm{th}}}.$$

The intensity of the measured contour in the probe image is known but its inner intensity remains inaccessible due to the binary nature of the recorded data. Assuming that an attenuation of the beam by using neutral density filters with factors *C*_i_, does not change the STID at focus, we can overcome this limitation. Repeating measurements with an additional attenuation by the factor *C*_i_ of the laser pulse yields a contour that encloses a smaller area because *I*_th_ is unaltered. Scaling this contour back to the full intensity of the pulse results in a scaled edge contour intensity:

2 $${I}_{{\rm{seci}}}(x,y,t)={I}_{{\rm{th}}}\cdot {C}_{{\rm{i}}}.$$

**Figure 4:**
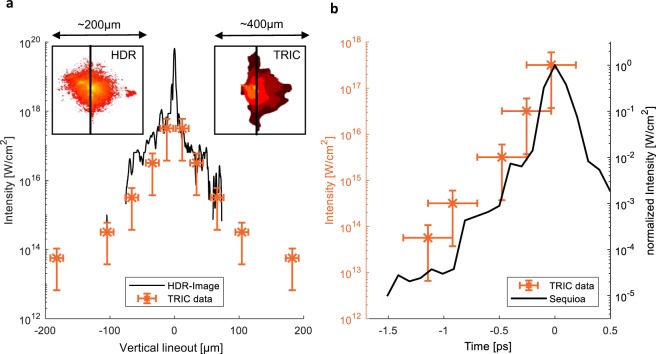
Comparison of TRIC to contrast curve and HDR focus image. (**a**) Compares the intensity distribution measured with TRIC and with a HDR camera. Line-outs of the HDR focus (left inset) and the TRIC focus (right inset) are shown. (**b**) Shows the temporal contrast of TRIC at *x* = 0 µm and *y* = 15 µm. For comparison the right axis shows a normalization compared to the measurement taken with the Sequoia-autocorrelator.

Repeating this for several *C*_i_ yields the absolute spatio-temporal intensity map as shown in Fig. 3. Each row corresponds to a single-shot with different attenuation coefficient and thus the scaled contour corresponds to a different intensity *I*_seci_. The summation over those measurements enables the retrieval of the absolute spatial intensity distribution of the laser focus at distinct time steps and is shown in Fig. 3b. Figure 3b is thus the complete STID of a high-power laser pulse in the focal plane, measured at full pulse energy on target and represents our main result. We note that the horizontal dimension is still temporally blurred due to the chosen probing geometry as described before. For further comparison a line-out at *x* = 0 µm (central vertical line) is used. The intensity map features measuring the spatial intensity profiles at select time steps and reveals the temporal dynamics at a given position. The most interesting cases are the spatial distribution of the peak intensity (*t* = 0) and the temporal contrast at the central point (*x* = 0 and *y* = 0). We therefore evaluated *I*(*x*, *y*, *y* = 189 fs) as displayed in Fig. 4a, since it was the closest time step past the interaction with the peak of the main pulse (*t* = 0) and *I*(*x* = 0 µm, *y* = 15 µm, t) as displayed in Fig. 4b. Due to in its current form limited spatial resolution we could not resolve the central area <25 µm. Therefore the most intense measured contour represents $3.18\cdot {10}^{17}$ · W cm^−2^, which is orders of magnitude below the classically estimated peak intensity of $5.76\cdot {10}^{19}$ · W cm^−2^.

### Comparison to contrast curve and laser focus

To assess our method we compared the extracted temporal and spatial intensity distribution to data obtained by conventional methods, a contrast profile of a third-order autocorrelator (averaged over a small portion of the collimated beam - no spatial information) *g*(*t*) and a HDR image of the laser in the focal plane (integrated over time) *f*(*x*, *y*)^26^. Both of those standard measurements yield only relative information, which we scale to absolute values by calculating

3 $$I(x,y,t)={E}_{Laser}\cdot f(x,y)\cdot g(t),$$

where *E*_*Laser*_ = 374 mJ is the full laser pulse energy measured for each shot (see methods). The spatial shape function of the laser focus *f*(*x*, *y*) and the temporal laser contrast-curve *g*(*t*) are both normalized, $\int \,f(x,y)dx\,dy=1$ and $\int \,g(t)\,dt=1$. Note that this separation of the temporal and spatial profiles neglects effects of spatio-temporal coupling^44^ by definition.

### HDR focus picture

The HDR focus image is the integrated spatial distribution over the full duration of the laser pulse and can thus be normalized to *f*(*x*, *y*). Assuming a Gaussian profile for the temporal distribution of the laser pulse, with a full-width at half-maximum ${\tau }_{FWHM}$ = 30 fs (laser pulse duration), the spatial intensity distribution of a measured HDR laser focus at time *t* = 0 is

4 $$I(x,y,t=\mathrm{0)}={E}_{{\rm{Laser}}}\cdot f(x,y)\cdot \frac{1}{\sqrt{2\pi }\sigma },$$

with $$\sigma =\frac{{\tau }_{FWHM}}{2.35}$$.

Figure 4a compares a line-out of this function along the vertical dimension (at x = 0) with the result obtained by TRIC at *t* = 189 fs. The focus, measured offline with the camera, appears continuous since it has more data points, while TRIC contributes a small number of points (dictated by the number of shots with different attenuation coefficients). In the overlap region, both methods yield similar comparable results. With TRIC we can measure down to *I*_th_, which exceeds the capability of HDR imaging as this is limited by damage of the camera. With an increased spatial resolution of TRIC, we expect to resolve also the peak of the pulse such that a 7 orders of magnitude dynamic range seems feasible. The horizontal error bar originates from the readout of the diameter of the contour. The rather large error bar for the evaluated intensities is due to the error of the determination in the threshold intensity.

### The temporal intensity distribution

The Sequoia is a scanning third order autocorrelator and measures the temporal intensity contrast relative to the laser peak. Comparison to the absolute temporal intensity evolution at a fixed spatial position, as accessible with TRIC, requires more assumptions. We thus solely compare the autocorrelator contrast curve to a normalized curve measured with TRIC as indicated in Fig. 4b (right axis). We normalized both intensities such that the peak (*t* = 0) is equal to one. The extent of temporal error bars of TRIC is attributed to the time interval correlated to the width of the bandpass filters for each time step in addition to an increment from time delay uncertainty. The intensity error bars originate from the uncertainty of the determined damage threshold. The TRIC values follow the shape of the autocorrelation trace but indicate reduced temporal contrast. The reason for this disagreement between the autocorrelation trace and the trace extracted from TRIC is not clear but one of the interesting features that can be studied with TRIC. Spatio-temporal coupling^44^, which can have diverse origins, manifests in focus (the simplest example is pulse lengthening by a pulse front tilt). This is where TRIC can yield complementary information. It highlights the necessity of monitoring and controlling the laser contrast on target at full energy, especially in the focal plane. More detailed measurements with an improved spatial resolution will help to shed light on this interesting aspect.

## Summary and Discussion

By using a simple method of beam multiplexing we enable recording of nine images of the plasma evolution during the interaction of a high-power laser pulse with a thin solid density target in a single-shot. Our interpretation of the such obtained images in the framework of TRIC yields information about the absolute STID in the focus of a high-power laser pulse. We note though, that if the spatial intensity distribution on target was significantly steeper, for example in the case of a perfect Gaussian pulse, this interpretation can be misleading and the transversely streaming electrons could contribute or even dominate the observes expansion front. Determining the STID in focus at high peak intensity is specially interesting in combination with plasma mirrors^16^ or other nonlinear techniques^45^ that aim to manipulate the laser pulse close to target. TRIC gives access to the spatial intensity distribution at energies slightly above *I*_th_. We note that it requires still analytical effort to account for the horizontal dimension. Because the ignition of the plasma and thus the change of opacity is irreversible on ultra-fast timescales, TRIC only yields information of the rising slope of the laser pulse and therefore requires the assumption of an intensity distribution that rises monotonically over time. Two short prepulses with intensities around 10^14^ · W cm^−2^ and thus just above the threshold intensity occurring ≈600 ps prior to the main pulse have been identified in the laser-system using third-order autocorrelation. If they were focused to a similar spot as the main pulse, they would have reached 10^14^ · W cm^−2^ for the shots with highest energy of 374 mJ. Although exceeding the intensity threshold for TRIC, we have not observed a respective contour when probing at times as early as 100 ps before the main pulse. We note though, that the contour with a size of a few µm could have not been detected with TRIC in its current spatial resolution limit. The absence of any sign of target damage/modification due to these prepulses on the other hand reconfirms that the measurement is not disturbed by transverse plasma-expansion initiated by very early prepulses at sufficiently low intensity, since no plasma shape is recorded in the probe beam until 1.3 ps prior to the peak of the pulse as evidenced in the last line of Fig. 3. It remains important to keep in mind such potential sources of miss-interpretation though. For example when prepulses have much higher intensity such pre-expansion can cause a plasma that indeed expands and mimics a contour that is not related to the intensity contour any longer. Such prepulses, however, would be easily identified in autocorrelation measurements and would not require TRIC. Additional uncertainties can be caused by absorption or divergence of the probe pulse in the plasma. However, the close resemblance between the contour of the hole and the plasma shape that we observed, does not indicate that this is relevant for extracting the contour lines from the images. Improving this resolution to a few µm will increase the sensitivity to prepulses just above the *I*_th_. A more detailed determination of the threshold intensity would further allow a reduction of this systematic error and thus an even more accurate determination of the absolute intensity distribution on target.

The technique of acquiring multiple images by exploiting the natural features of a broadband highly intense laser-pulse, can be adapted to other, more complicated probing techniques, e.g. using holography^46,47^ instead of binary shadowgraphy. It can therefore add a temporal component to diverse probing experiments. Thus, we see TRIC as a very first step that is accessible with this technique. In the future we foresee a potential use of TRIC for different pump-probe modalities. Especially the intrinsic synchronization in laser-driven particle acceleration processes enables temporal resolved investigations of the interaction of ions, electrons and x-rays with matter. The demonstrated sub-ps temporal resolution could for example also be exploited further for development of a time-of-flight measurement^48,49^ of ion bunches with combined temporal and 2D spatial resolution.

## Methods

The ATLAS-300 is a single CPA laser system based on a Titanium Sapphire system and can be operated at a 5 Hz repetition rate. The pulse length of 30 fs is measured with FROG^31^ and the temporal contrast was measured with a third-order autocorrelator (Sequoia or Tundra). Several Pockels cells are implemented in the system to clean the ns contrast, the fastest one has a rise time of about 500 ps. The temporal laser contrast has been found to be in the order or 10^−9^ from a few ns up to shortly before the main pulse ≈50 ps measured over 2 ns prior to the main pulse. Two short prepulses with ≈10^−6^ of peak power have been identified about 600 ps prior to the main pulse, originating from reflections in the regenerative amplifier. Further measurements with saturated photodiodes have not shown any significant prepulses in the ns range prior to the pulse. Therefore the majority of the energy is in the main pulse and the measured energy (determined with an energy meter) is used for calculations of the focus intensity. To monitor the energy of each shot a second energy meter is positioned behind a leakage mirror close to the experiment. At lower energies the sensitivity of the energy meter was not sufficient. In this case, the energy was estimated by integrating over the spectrum measured with an Ocean Optics spectrometer for each shot. The neutral density filters that are used to attenuate the beam during the experiment are positioned in the stretched beam before the compressor (≈400 ps). The probe beam is coupled out by a pickoff mirror about 2 meters before the laser-target interaction at the edge of the beam. The main laser is focused with a 90°, *f* = 2 silver parabola with 20 cm focal length impinging onto target with p-polarization. The resulting HDR focus picture is depicted in Fig. 3 with a resulting FWHM of about 3 µm. The HDR image of this time-integrated focal spot distribution provides the basis for calculating the peak intensity^26^.

**The probe pulse** was coupled out with a pick off mirror at the edge of the main laser pulse in the target chamber (spatial sampling). The timing of the probe beam with respect to the main laser pulse was controlled with a delay stage. The probe beam was then guided through an aperture of 7.5 mm diameter. This aperture was imaged onto the interaction point (IP) with a demagnification of 9, resulting in an illuminated area of ≈830 µm. A glass rod of 3 cm length was introduced in the beam path prior to target in order to chirp the pulse up to 2 ps. The energy of the probe beam was ≈0.5% of the energy of the main pulse, resulting in a maximum of 20 mJ and an intensity of 1 · 10^12^ · W cm^−2^ on target. The intensity of the probe was thus 5 orders of magnitude lower than the intensity of the main pulse and further distinctly smaller than the threshold intensity. Due to the modified energy of the laser and thus also the probe beam, it was further filtered in front of the camera (for high energies). An intended good contrast between the image created by the probe and self-emission from the target favored filtering in front of the camera instead of filtering prior to the target. The imaging system of the probe beam was based on a two-lens-system resulting in a magnification of the probe image (in focal plane) of ≈6.5. The first lens (2 inch) with a focal length of 10 cm is used to collect the light so it can thus further be guided out of the vacuum chamber. A second lens is used to image the IP onto the camera. A transmission grating (Collischon, 15 µm lattice constant) is placed in the beam path of the second lens to the camera, creating 9 nearly identical replicas on the camera chip. The narrow bandpass (10 nm FWHM) filters with dimensions 5 × 5 · mm^−2^ (Omega Optical Inc.) are placed directly in front of the camera chip. The spatial resolution of the complete setup was determined experimentally to about 25 µm. This is significantly lower than the theoretical resolution of the imaging system, which would support ≈2 µm (Abbe limit). The main limiting factor is the low quality of the beam profile of the probe beam, which shows strong intensity fluctuations on the order of 10 µm spatial scale.

**The zero timing** describes the coincidence of the incident of probe and main pulse on the target. It was measured with the use of an air plasma ignited by the attenuated pump pulse in air. A high-power laser pulse can generate an air plasma when a certain intensity threshold^50^ is reached. Therefore the laser intensity was diminished until the air plasma was solely visible in one or two of the nine frames and therefore marking the peak of the laser pulse with an accuracy of ±222 fs. It should be noted that the probe beam is coupled out in the vacuum chamber and has thus also been in air for the determination of the zero timing.

**The frame rate** of the camera is set by the temporal spacing of the spectral images and the chirp of the probe pulse. Therefore the group velocity dispersion has to be calculated including complete knowledge of the stretching material in the probe beam and respective material characteristics. Since multiple error sources are introduced, we chose a different approach. Two nine-frame images are created, differing in delay by 1000 steps of the motor of the delay stage. If a specific plasma size is observed in two different frames of both nine-frame images, one can check for the change in the frame number and unambiguously correlate frame-number and temporal delay. Knowing that 1000 steps equal a delay of 666 fs the frame rate can be simply calculated. In the setup described above, a 3 cm glass rod resulted in a delay of 222 fs between each frame and a total observation time of 2 ps within a single-shot.

**The threshold intensity** was determined by focusing the main laser pulse onto a 200 nm thick plastic foil with a peak intensity that was calculated via equation 4. The target was examined after the shot with a 20 times magnifying microscope in the vacuum chamber. By reducing the intensity stepwise according to the values given in Fig. 2 we identified that the target had no visible hole after a single-shot with peak intensity ${I}_{7}=5.77\cdot {10}^{13}$ · W cm^−2^, whereas a clear hole was visible after a shot with *I*_6_. We thus, supported by the extensions of the hole in the target after one shot with *I*_6_, determined the threshold intensity of the 200 nm plastic target to be ${I}_{{\rm{th}}}=\mathrm{(6}\pm \mathrm{5)}\cdot {10}^{13}$ · W cm^−2^. We further note, that we empirically found that the intensity, needed to create an overdense and thus opaque plasma, also caused a hole in the target. The laser-induced damage threshold of different materials has been discussed widely over the last decades. The correlation between the conduction band (free) electron density reaching the critical density and damage due to ablation, is broadly accepted^51,52^. However, newer research indicates that higher electron densities are required until damage occures^53,54^. Because we only use the transition from transparent to opaque for determining the contours, which happens for any free electron density *n*_*e*_ ≥ *n*_*c*_, our method is not sensitive to the exact damage mechanism. The threshold intensity ${I}_{{\rm{th}}}=\mathrm{(6}\pm \mathrm{5)}\cdot {10}^{13}$ · W cm^−2^ should therefore not be associated with a damage threshold. Note, that the large error bar of *I*_th_ results from the course intensity filtering steps.

## Data Availability

The datasets generated during and/or analyzed during the current study are available from the corresponding author on reasonable request.
